# Free Radical Scavenging, Anti-Infectious, and Toxicity Activities from *Stenochlaena palustris* (Burm.f.) Bedd. Extracts

**DOI:** 10.1155/2022/5729217

**Published:** 2022-11-01

**Authors:** Rudi Hendra, Rohimatul Khodijah, Muhammad Almurdani, Yuli Haryani, Ari Satia Nugraha, Neni Frimayanti, Hilwan Yuda Teruna, Rizky Abdulah

**Affiliations:** ^1^Department of Chemistry, Faculty of Mathematics and Natural Sciences, Universitas Riau, Pekanbaru, Indonesia; ^2^Center of Excellence in Pharmaceutical Care Innovation, Universitas Padjadjaran, Bandung, Indonesia; ^3^Sekolah Tinggi Ilmu Farmasi Riau, Pekanbaru, Indonesia; ^4^Drug Utilisation and Discovery Research Group, Faculty of Pharmacy, University of Jember, Jember, Indonesia; ^5^Department of Pharmacology and Clinical Pharmacy, Faculty of Pharmacy, Universitas Padjadjaran, Jatinangor, Indonesia

## Abstract

*Stenochlaena palustris* (Burm.f.) Bedd. *(Blechnaceae)* or Kelakai is a traditional medicinal plant found in the Indonesian islands of Borneo and Sumatra. It has been used to treat wound healing, infection, and diabetes. This study looked into the free radical scavenging activity, antiplasmodial activity, toxicity, and antibacterial activity against pathogenic bacteria. The species' aerial part was extracted with methanol, followed by a liquid-liquid extraction against (*n*-hexane, dichloromethane, and ethyl acetate). The extracts' free radical scavenging activities were determined using DPPH and NO radicals. The antiplasmodial and toxicity assays were conducted using two *Plasmodium falciparum* strains (3D7 and W2) and the brine shrimp lethality test. In addition, antibacterial activity was determined using the well diffusion method. The results revealed that ethyl acetate depicted potential activities toward the assay. The ethyl acetate showed potential free radical scavenging activities with an IC_50_ value of 51.63 ± 0.46 *μ*g/mL (DPPH) and 60.03 ± 0.65 *μ*g/mL (NO). The antiplasmodial activities showed that the ethyl acetate had potential activities among the extracts with an IC_50_ value of 11.06 ± 0.45 *μ*g/mL. However, all the extracts demonstrated nontoxic toward *Artemia salina* with LC_50_ > 1000 *μ*g/mL. Furthermore, the ethyl acetate demonstrated intermediate susceptibility against *B. cereus* ATCC 10876, *V. parahaemolyticus* ATCC 17802, *L. monocytogenes* ATCC 7644, and *S. Typhimurium* ATCC 14028 at a concentration of 500 *μ*g/disc. According to these findings, the ethyl acetate extract of *S. palustris* (Burm.f.) Bedd is a promising source of natural antioxidants and antiplasmodial agents.

## 1. Introduction

Recent years have seen an increase in people's interest in traditional medicine as a result of its multiple modes of action, which have relatively few negative effects on humans. A plant with medicinal properties is a rich source of secondary metabolites, which are used in a variety of treatments. Because of this and considering the high level of biodiversity that exists in Indonesia, it is imperative that potential plant species, including ferns, be studied [1]. Several groups have conducted pharmacological research on ferns and related plants in response to reports of their therapeutic efficacy, scientific curiosity, and the need for new medications. Pharmacological and ethnopharmacological studies of fern substances have revealed various pharmacological effects, including cytotoxicity, hepatoprotective activity, anti-inflammatory activity, and antimicrobial properties. Due to the need for new medications with such activities, pteridophytes and their secondary metabolites may have considerable medical value [2].

A fern belonging to the family *Blechnaceae*, *Stenochlaena palustris* (Burm.) Bedd. can be found scrambling high up trees or trailing along the ground. It is native to many tropical regions, including southern and northern India, Malaysia, Polynesia, and Australia [3]. This particular species belongs to the family *Blechnaceae*, which contains seven species, including one endemic in the Province of Riau, *S. riauensis* [4]. In Malaysia, Thailand, the Philippines, and Indonesia, the reddish young fronds are harvested from the wild and consumed as vegetables [5].

This species has been used in traditional medicine in some countries to treat a variety of ailments. People in the Western Ghats of India, for example, use this species' fronds to treat fever, sore throat, and gastric ulcers. While the leaves and rhizomes are used as a cooling agent in the treatment of burns and ulcers [6]. In Malaysia, the plant is consumed raw or cooked with boiling water for the treatment of diarrhea, while in Sumatra, Indonesia, it is used as a mild laxative [7]. In addition, the paste is applied to ulcers, wounds, and bacterially infected skin in Sumatra Island, Indonesia, Borneo Island, Malaysia, and Indonesia. People in the central region of Papua New Guinea use the tender leaves of *S. palustris* (Burm.f.) Bedd. as a contraceptive and the plant's juice can aid in the treatment of fever [5, 8].

Although this species is edible and has medicinal properties, several pharmacology and phytochemistry studies have been published. According to Chai et al., crude water extracts of *S. palustris* (Burm.f.) Bedd. had higher antioxidant activity and polyphenol, flavonoid, and hydroxycinnamic acid contents [7, 9]. Chai and colleagues also revealed that water extract from the species acted as a potent *α*-glucosidase inhibitor compared to quercetin as a control [10]. Furthermore, *S. palustris* (Burm.f.) Bedd. leaf extract inhibited the food-borne pathogen *Aspergillus Niger* [11]. In addition, the methanolic extract of this species showed cholinesterase inhibitory activity against acetylcholinesterase and butyrylcholinesterase with IC_50_ of 121 and 19.8 *μ*g/mL, respectively [12]. There is a paucity of information available regarding the phytochemistry of *S. palustris* (Burm.f.) Bedd. stenopaluside, cerebroside, lutein, and *β*-sitosterol-3-O-*β*-D-glucopyranoside were isolated from the leave of *S. palustris* (Burm.f.) Bedd [13]. In addition, stenopalustrosides A-E and kaempferol glucoside were isolated from species' leaves, and stenopalustrosides A-D showed significant antibacterial activities against Gram-positive strains [3]. Furthermore, kaempferol 3-O-(3″-O-E-*p*-coumaroyl)-(6″-O-E-feruloyl)-*β*-D-glucopyranoside and kaempferol 3-O-(3″, 6″di-O-E-p-coumaroyl)-*β*-D-glucopyranoside isolated from the species showed cytotoxic properties against breast (MCF-7 and MDA-MB-231) and prostate (DU-145) cancer cell lines [14].

In this study, we reported the details of the extraction of *S. palustris* (Burm.f.) Bedd. producing less polar subextracts, as well as the free radical scavenging evaluation. In a previous study according to the findings of Arullappan et al., ethyl acetate extracted from the leaves of *S. palustris* (Burm.f.) Bedd. exhibited DPPH radical inhibitor activity with an IC_50_ value of 650 *μ*g/mL [5]. Even though the DPPH method is used generally in antioxidant activity, the results are not standardized in which direct comparison of antioxidant strength in both different plant extracts and pure compounds are difficult. Therefore, in this study, we report the antioxidant activity index based on the method by Scherer and Godoy (2009) [15]. This analysis allows us to compare the antioxidant capacity of the extracts to that of the positive control. Moreover, this is the first time that radical scavenging activity using the nitric oxide radical that correlates with inflammation has been reported from the extracts of this species. A novel antiplasmodium and antibacterial activity from the species' extract for the first time. In addition, molecular chemotypes previously generated from this species were also discussed in correlation with the planta pharmacological claims.

## 2. Materials and Methods

### 2.1. Plant Material

Fresh fronds of *S. palustris* (Burm.f.) Bedd. were gathered at Science Park Universitas Riau (0.46587747854712624 and 101.38066586137639). Before the grinding process began, the species was air-dried and confirmed to be completely dry, as its weight remained constant. The soil samples were then placed in an airtight container and stored between 2 and 7°C until further analysis. A botanist (Dr. Nery Sofianty) has deposited a voucher specimen (No. 37/UN19.5.1.1.3/2019) with the Department of Biology at Universitas Riau.

### 2.2. Extraction

Maceration in methanol of up to 2 kg of ground samples for 1 × 24 hours was repeated three times until the maceration results were no longer dark green. The extract was filtered during the maceration process, and the filtrate was collected. The macerate was then concentrated using a 40°C rotary evaporator, yielding a crude extract. The crude methanol extract was then partitioned using a liquid-liquid extraction method. To obtain the *n*-hexane extract, the methanol extract was partitioned with water in a 9 : 1 methanol: water ratio before being partitioned with *n*-hexane in a 1 : 4 ratio. The methanol-water extract was then watered down to 40% (v/v) and partitioned with dichloromethane solvent in a 1 : 4 ratios to produce dichloromethane extract. The methanol-water extract was then evaporated to form a water extract, which was then partitioned in a 1 : 3 ratio with ethyl acetate to yield the ethyl acetate extract. The remaining water extract was evaporated to produce the finished product, which was water extract [16].

### 2.3. Antioxidant Activities

#### 2.3.1. DPPH Radical Scavenging Activity

An antioxidant activity assay was carried out using the DPPH method (1,1-diphenyl-2- picryl hydrazyl). [1, 17, 18] Various extracts with a final concentration of 1000 *μ*g/mL were diluted using the two-fold dilution method (1000–31.25 *μ*g/mL) in a 96-well clear polystyrene microplate. Fifty *μ*L of the sample was mixed with 80 *μ*L of 80 *μ*g/mL DPPH, and the mixture was incubated in the dark for 30 minutes. Absorbance was measured at 520 nm using a Berthold microplate reader. As a positive control, ascorbic acid was subjected to the same method. The assay was carried out in triplicate, and the data were reported as mean ± standard deviation.

The % Inhibition value is calculated by the following formula:

(1) $$\begin{array}{c} \%\text{Inhibition}=\frac{\left(A_{0}-A_{s}\right)}{A_{0}}\times 100\%\text{.} \end{array}$$

IC_50_ analysis was performed using GraphPad Prism 9 software, where *A*_*0*_ is the absorbance of the DPPH radical solution without the sample and *A*_*s*_ is the absorbance of the sample with DPPH radical solution. The assay was carried out in triplicate, and the data were reported as mean ± standard deviation.

The antioxidant activity index (AAI) is calculated as follows:

(2) $$\begin{array}{c} \text{AAI}=\frac{\text{DPPH}\left(\mu g/mL\right)}{IC50\left(\mu g/mL\right)}\text{.} \end{array}$$

#### 2.3.2. Nitric Oxide Scavenging Activity

The nitric oxide (NO) scavenging activity of various extracts was determined by Oskoueian et al. [19]. In a 96-well flat-bottomed plate, sixty microliters of the sample that had been diluted two-fold were combined with sixty microliters of sodium nitroprusside dissolved in phosphate-buffered saline (PBS) at a concentration of ten millimoles per liter. This mixture was then incubated at room temperature for 150 minutes while exposed to light. In the end, an equal volume of the Griess reagent was added to each well to measure the NO content. Ascorbic acid was used as a positive control in this experiment. The NO scavenging activity was calculated according to the formula: [(*A*0 − *A*1)/*A*0] × 100%, where *A0* was the absorbance of the control reaction and *A1* was the absorbance in the presence of the sample. The assay was carried out in triplicate, and the data were reported as mean ± standard deviation. IC_50_ analysis was performed using GraphPad Prism 8 software.

### 2.4. *In Vitro* Antiplasmodial Assays

In this analysis, chloroquine-sensitive (3D7) and chloroquine-resistant (W2) strains of *Plasmodium falciparum* were utilized. A total of 1 mg of the sample was dissolved in 100 *μ*l of DMSO (stock solution, 10,000 *μ*g/ml concentration), and a serial dilution was made from the stock solution. The parasites used in this test were synchronous (ring stage) parasites with less than one percent parasitemia. Then, 198 *μ*l of parasite was added (the final concentrations of test material were 100 *μ*g/ml, 10 *μ*g/ml, 1 *μ*g/ml, 0.1 *μ*g/ml, and 0.01 *μ*g/ml). The plate was placed within the chamber, and gas was blended (O_2_ 5%, CO_2_ 5%, and N_2_ 90%). Forty-eight hours were spent incubating the chamber containing the plate at 37°C. The culture was then harvested, and a 20% Giemsa-stained blood layer formed. Under a microscope, the number of infected erythrocytes per 1000 healthy erythrocytes was determined for the performed blood test. DMSO was used as a negative control and artemisinin as a positive control (standard drug) to avoid false results. Then, the percentage of growth and inhibition was determined by analyzing the data. Based on the percent inhibition results, a statistical analysis using a probit analysis in Minitab®19 was conducted to determine the IC_50_ value or concentration of the test material that could inhibit the parasite's growth by up to 50%. The assay was carried out in triplicate, and the data were reported as mean ± standard deviation.

### 2.5. Toxicity Assay

The brine shrimp lethality test was utilized to determine the toxicity level exhibited by the extracts (BSLT). After preparing ten vials, each containing 2 mL of seawater, for the purpose of conducting the test method, a two-fold dilution was performed to generate several different concentrations. After applying an aliquot (0.1 mL) containing approximately ten nauplii to each vial, the setup was allowed to continue for twenty-four hours. After twenty-four hours, the contents of each bottle were examined, and dead larvae were tallied. As a form of negative control, dimethyl sulfoxide, or DMSO, was utilized. The assay was carried out in triplicate, and the data were reported as mean ± standard deviation. By plotting the median mortality percentage against the log of concentration, we could determine the concentration (LC_50_) that resulted in fifty percent of the population dying as a direct result of being exposed to the extracts.

### 2.6. Antibacterial Activity

#### 2.6.1. Microbial Strain

Seven microorganisms, namely, *Bacillus cereus* (ATCC 10876), *B. subtilis* (ATCC 19659), *Escherichia coli* (ATCC 8739), *Listeria monocytogenes* (ATCC 7644), *Staphylococcus aureus* (ATCC 6538), *Salmonella typhimurium* (ATCC 142028), and *Vibrio parahaemolyticus* (ATCC 17802) were used in this study. The microbial isolates were maintained on an agar slant at four °C in the Laboratory of Biochemistry, Department of Chemistry, Universitas Riau. Before any antimicrobial test, the strains were subcultured for 24 hours on a fresh agar plate.

#### 2.6.2. Antibacterial Activity

Subsequently, 100 *μ*L of nutrient broth containing pathogenic bacteria was inoculated in nutrient agar. The extracts were screened for their antibacterial activity against those pathogenic bacteria by using the agar-disc diffusion method with a cell suspension of about 1.5 × 106 CFU/ml obtained from a McFarland turbidity standard No 0.5. A 6 mm sterile filter paper disc was immediately placed into the inoculated media, and each disc was filled with 20 *μ*L extracts with a final concentration of 500 *μ*g/disc with chloramphenicol (30 *μ*g/disc) as a positive control, incubated overnight at 37°C. After 24 hours of incubation, the inhibition zone (in mm) was measured. The diameter (in millimeters) of the inhibition zone surrounding the well was measured using a caliper. All procedures were carried out in triplicate. Minitab®19 was used to analyze mean comparisons, and the data were presented as mean ± SD.

### 2.7. Statistical Analysis

All tests were performed in triplicate and the obtained results are expressed as mean ± SD. Data were analyzed using GraphPad Prism Version 9, and comparisons of the means were determined by one-way analysis of variance (one-way ANOVA) followed by the Tukey test. Values were significantly different if they fall below the significance level (0.05).

## 3. Results

### 3.1. Radical Scavenging Activity

This study's free radical scavenging activities were determined by using two chemical assays, namely, DPPH and nitric oxide radicals. The results showed that there was a significant difference between the extracts and the ethyl acetate extract depicted the highest inhibition toward DPPH radical with a percentage of inhibition value of 96.87% ± 0.67, followed by dichloromethane at 95.21% ± 0.37 at a concentration of 1000 *μ*g/m, while the *n*-hexane extract had the lowest inhibition at 62.21% ± 1.09.

In addition, the extracts' half-maximal inhibitory concentration (IC_50_), along with ascorbic acid serving as a positive control, was evaluated in GraphPad Prism 9 using a dose-response curve (Table 1). The findings demonstrated that the ethyl acetate extract had the lowest IC_50_ among the extracts, with a value of 51.63 ± 0.46 *μ*g/mL. Despite this, the findings showed that the ethyl acetate extract was still significantly less potent than ascorbic acid, which had an IC_50_ of 4.15 ± 0.24 *μ*g/mL. In addition, the extracts' antioxidant activity index displayed a wide range of activity. According to Table 1, dichloromethane and ethyl acetate extracts have the highest antioxidant activity index, whereas *n*-hexane extracts have the lowest index. Even though ethyl acetate and dichloromethane had the highest index, the activity of these two compounds was regarded as being inferior to that of ascorbic acid.

Moreover, the radical scavenging activity of the extracts was examined concerning the nitric oxide (NO) radical, and the results showed that the extracts inhibited the NO radical in a manner dependent on the dose. On top of that, dichloromethane and ethyl acetate demonstrated high activity levels among the extracts, with their respective percentages of inhibition emerging at 83.40 ± 0.45% and 85.17 ± 0.58%. Furthermore, the IC_50_ value of the extracts was analyzed in the same manner as IC_50_ for the DPPH assay. The ethyl acetate extract showed high activity with IC_50_ 60.30 ± 0.65 *μ*g/mL, where the *n*-hexane possessed the lowest activity (956.70 ± 0.54 *μ*g/mL), and compared to the ascorbic acid, the extracts showed low activity toward NO radical.

### 3.2. *In Vitro* Antiplasmodial and Toxicity Assays

The antiplasmodial activity against chloroquine-sensitive 3D7 and chloroquine-resistant W2 strains of *P. falciparum* was evaluated in vitro, as was the toxicity using the brine shrimp lethality test. Each of the four extracts exhibited activity against *P. falciparum* in a dose-dependent manner and at 100 *μ*g/mL; ethyl acetate inhibited the growth of *P. falciparum* by 69.85 ± 0.22%, while dichloromethane extract showed a comparable activity level at 64.18 ± 0.22%. The activity level observed with the ethyl acetate was about twice higher as the dichloromethane with IC_50_ of 11.06 ± 0.45 and 21.68 ± 0.56 *μ*g/mL, respectively. However, these results show that the extracts exhibited lower activity than artemisinin, with an IC_50_ value of 0.0054 ± 0.0013 *μ*g/mL. Furthermore, the extracts were applied with a brine shrimp lethality test to their toxicity level, and the findings showed that the extracts exhibited similar toxicity levels (Table 2).

### 3.3. Antibacterial Activity

The effect of the crude extracts on the growth of seven tested indicator bacteria is shown in Table 3. Ethyl acetate extract exhibited the best extract as it presented the highest inhibition against *B. cereus* ATCC 10876, *V. parahaemolyticus* ATCC 17802, *L. monocytogenes* ATCC 7644, and *S. Typhimurium* ATCC 14028, followed by the dichloromethane extract with greatest clear zone against *B. subtilis* ATCC 19659, and *S. aureus* ATCC 6538. The minor activity was shown by the *n*-hexane extract, and none of the crude extracts affected the growth of *E. coli* ATCC 10876.

## 4. Discussion

This study applied the solvent-solvent partition technique with a modified Kupchan partition to the methanol extract [20]. Separating a total MeOH extract from four large extracts, which are simplified mixes of different polarities, using only partitions made from nonmiscellaneous solvents, is the main objective of the Kuphan partition, a practical and straightforward method for initiating a purification procedure. It is possible for there to be significant differences in distribution between extracts of compounds with varying degrees of polarity [17], and then, the extracts were investigated for their antioxidant, antiplasmodial, and antibacterial properties.

An antioxidant is a substance that significantly retards or prevents the oxidation process. The antioxidant activity is indirectly measured by determining the rate at which oxidation processes are inhibited in the presence of an antioxidant. [21] Each extract was then analyzed for its antioxidant activity using the free radical (DPPH) scavenging method. DPPH (2, 2-diphenyl-1-picrylhydrazyl), an organic stable radical in crystalline form and solution, is often used to measure a compound's antiradical activity or extract. The ability of a compound or extract to get rid of free radicals is often linked to its ability to act as an antioxidant. [22].

Despite the widespread use of the DPPH method, the lack of standardization of the results makes it difficult to compare the antioxidant potency of various plant extracts and pure compounds. Scherer and Godoy, therefore, proposed a new antioxidant activity index (AAI). The AAI was calculated based on the mass of DPPH and the mass of the sample in the reaction, resulting in a constant for each sample regardless of the DPPH concentration and sample used. The sample is considered to have a low level of antioxidant activity when the antioxidant index (AAI) is less than 0.50. When the AAI falls between 0.5 and 1.0, the sample's antioxidant activity is considered moderate. When the AAI is between 1.0 and 2.0, it is considered that the sample has a high level of antioxidant activity. [15] According to these standards, the ethyl acetate and dichloromethane extracts should be considered to have a high level of antioxidant activity.

The radical scavenging activities of these extracts might be the presence of secondary metabolites, specifically phenolics, and flavonoids. Flavonoids might have multiple properties for removing reactive oxygen and nitrogen species. Typically, the presence of an ortho-hydroxylation on the B-ring of the flavonoid molecule, the number of free hydroxyl groups, a C2-C3 double bond in the C-ring, or the presence of a 3-hydroxyl group is listed as a condition of antioxidant and antiradical activity. [23] According to findings from earlier research, *S. palustris* (Burm.f.) Bedd. contains acylated and nonacylated kaempferol glycosides, each of which possesses free radical scavenging activity toward the DPPH radical at IC_50_ concentrations ranging from 120 to 400 *μ*M. [14] On the basis of these findings, it is possible to conclude that the extracts of *S. palustris* (Burm.f.) Bedd., more specifically dichloromethane and ethyl acetate, can inhibit free radicals. These findings are consistent with a few other publications that have been done in the past. According to the findings of Arullappan et al., ethyl acetate extracted from the leaves of *S. palustris* (Burm.f.) Bedd., which originated in Malaysia, exhibited DPPH radical inhibitor activity with an IC_50_ value of 650 *μ*g/mL. In addition, the ethyl acetate extract of *S. palustris* (Burm.f.) Bedd. from Perak, Malaysia, demonstrated a high level of radical scavenging activity, and its IC_50_ concentration was found to be 49.8 *μ*g/mL, which is comparable to the results found in the report [5, 10].

Furthermore, to explore the potential of the various extracts toward free radicals, nitric oxide (NO) inhibitor was determined. Nitric oxide, also known as NO, is a potent pleiotropic mediator produced from the amino acid L-arginine by vascular endothelial cells, phagocytes, and specific brain cells. When NO combines with the superoxide radical, a highly reactive peroxynitrite anion known as ONOO^−^is produced. This is when NO's toxicity turns for the worse [24]. The reaction between the nitric oxide produced by sodium nitroprusside and oxygen results in the formation of nitrite. The nitrite ions diazotize with sulfanilic acid and couple with naphthyl ethylenediamine, forming a pink color with a wavelength of 546 nm. [25] Table 1 shows the corresponding IC_50_ values for NO scavenging activity, and the dichloromethane and ethyl acetate extracts had IC_50_ values less than 200 *μ*g/ml, indicating good NO-scavenging activity, whereas the *n*-hexane and water extracts had IC_50_ values greater than 400 *μ*g/ml, indicating poor NO scavenging activity. The NO scavenging values were classified based on Tsai et al. [26].

Moreover, the free radical scavenging activity of *S. palustris* (Burm.f.) Bedd. extracts, specifically the dichloromethane and ethyl acetate against the nitric oxide (NO) radical, showed suitable activities. This is due to the presence of kaempferol glycoside within species. Multiflorins A, a kaempferol glycoside, isolated from fern *Neocheiropteris palmatopedata* showed a percentage inhibition of NO 52% at 20 *μ*g/ml, and kaempferol was also reported to inhibit the production of NO in RAW 264.7 cells line at IC_50_ 90.3 *μ*M [27, 28].

Oxidative stress is thought to play a role in developing several diseases, including cancer, diabetes, and renal disease. This stress is brought on by the excessive production of nitric oxide (NO), which can result from infection or inflammation. As a result, removing NO radicals or inhibiting NO production by mitogen-activated cells may be promising indicators for discovering new treatments for those ailments. [26] Consequently, as a result of these studies, the extract of this species can further have its potential inhibition in the cell line of animal studies investigated. The presence of a NO inhibitor originating from this species has not previously been documented. As a consequence of this finding, additional information regarding the free radical scavenging activities of the species is provided.

In addition, it was discovered that all four extracts possessed varying degrees of activity against chloroquine-sensitive 3D7 strains of *P. falciparum* and chloroquine-resistant W2 strains (Table 2). According to the WHO guidelines and the fundamental requirements of antiparasitic drug discovery, the activities of the extracts were separated into four categories based on their values for the IC_50_ concentration: high activity (IC_50_ ≤ 5 *μ*g/ml), promising activity (5 *μ*g/ml < IC_50_ ≤ 15 *μ*g/ml), moderate activity (15 *μ*g/ml < IC_50_ ≤ 50 *μ*g/ml), and poor activity (IC_50_ > 50 *μ*/ml). [29, 30] Therefore, the dichloromethane and ethyl acetate extracts have a moderate and promising antiplasmodial activity, respectively, whereas the *n*-hexane and water extracts were classified as having a poor activity. This might be due to the presence of kaempferol glucoside, such as kaempferol 3-O-*α*-L-rhamnopyranoside, isolated from *S. palustris* (Burm.f.) Bedd. leave. This compound has been reported that possessed promising antiplasmodial activity against chloroquine-resistant *P. falciparum* with an IC_50_ of 106 *μ*M [14, 31]. This finding is supported by a previous report that determined the antimalarial activity of an ethanol extract of *S. palustris* (Burm.f.) Bedd. leaves against parasitemia and splenomegaly of *Plasmodium be*rghei ANKA in infected BALB/c mice. The results indicated that the extract possessed good antimalarial activity at an ED50 value of 77.05 mg/kg body weight [32].

The extracts were put through a lethality test using brine shrimp to determine their overall level of toxicity. The test is a straightforward and highly effective method for determining the toxic potential of bioactive substances or extracts. It is based on the ability of test compounds to kill shrimp, a relatively simple zoological organism (*Artemia Salina*) [33]. According to Meyer and the coauthor, the plant extracts demonstrated toxicity with an LC_50_ value of less than 1000 *μ*g/ml; however, the plant extracts were regarded as highly toxic for the shrimp with an LC_50_ value of less than 30 *μ*g/ml [34]. The findings showed a nontoxic LC_50_ value of >1000 *μ*g/ml for all the extracts (Table 2). This result contradicts the previous report, which mentions that *S. palustris* (Burm.f.) Bedd. ethanolic extract growth in Bangladesh showed the LC_50_ at 184.7 *μ*g/ml using the BSLT method. [35] The toxicity of brine shrimp has been found to predict cytotoxicity and parasite activity. There was a strong correlation between BSLT toxicity and cytotoxicity against the 9 KB cell line (human nasopharyngeal carcinoma) and other tumors and *in vivo* murine leukemia. The BSLT detects potent anticancer activity, but its predictive ability to distinguish between potent, moderate, and weak anticancer compounds is limited. Thus, the BSLT provides a rapid screening for potent cytotoxins and a higher level of cancer discrimination. [36].

In general, the majority of the tested extracts exhibited weak antibacterial activity. As shown in Table 3, extracts at 500 *μ*g/disc exhibited variable inhibitory activity against all bacteria, with inhibition zone diameters ranging from 0.7 to 1.5 cm. The responses of microorganisms to the different extracts varied. In this study, it was determined that all extracts inhibited Gram-positive bacteria more effectively than Gram-negative bacteria. As supported by these results, Gram-negative microorganisms are typically more resistant to antimicrobial agents than Gram-positive bacteria. This has long been attributed to an outer-membrane permeability barrier in Gram-negative bacteria, which prevents antimicrobial agents from reaching their targets within the bacterial cells [37]. Even though the antibacterial activity from the extract exhibited weak antibacterial, it is noteworthy that the finding from Liu et al. showed that stenopalustrosides A-D showed significant antibacterial activities against Gram-positive strains (*Bacillus cereus, Staphylococcus epidermidis, S. aureus, and Micrococcus luteus*) with MIC between 2 and 64 *μ*g/mL [3]. Another report showed that methanol extract from *S. palustris* (Burm.f.) Bedd. exhibited various antibacterial activities against the pathogenic bacteria with a MIC range between 50 and 12.5 mg/mL [8].

The species, on the whole, demonstrated a wide range of biological activities throughout the subject of this investigation. According to the findings of the study, the species exhibit a potential activity by scavenging free radicals (DPPH and nitric oxide), and the ability to inhibit nitric oxide radicals in this species was reported for the first time. Aside from that, it was the first time that the antiplasmodium activity of this species was reported, and it has a good potential to inhibit the growth of both strains of *plasmodium*. On the other hand, this particular species does not exhibit significant antibacterial activity. The limitation of this study, however, is that we did not report the phytochemical compounds present in this species. Instead, we correlated the biological activities revealed in this study with compounds already known to be present in the species based on previously published research.

## 5. Conclusions

The results of this study have demonstrated that an extract of *S. palustris* possesses promising antioxidant and antiplasmodial activities. The dichloromethane and ethyl acetate extracts showed significant levels of antiplasmodial and radical scavenging activities. The antibacterial activities of the extracts, on the other hand, showed only a weak level of activity against pathogenic bacteria These findings provide a basis for further investigation into the isolating and analyzing the biological activity of secondary metabolites found in the extracts.

## Figures and Tables

**Table 1:** The IC_50_ values of various extracts of *S. palustris* (Burm.f.) Bedd. in DPPH and nitric oxide scavenging activities.

Sample	*DPPH*		Nitric oxide
	IC_50_ (*μ*g/mL)	Antioxidant activity index (AAI)	IC_50_ (*μ*g/mL)
*n*-Hexane extract	233.60 ± 0.54	0.34 ± 0.044	956.70 ± 0.54
Dichloromethane extract	58.33 ± 0.24	1.37 ± 0.065	83.44 ± 0.22
Ethyl acetate extract	51.63 ± 0.46	1.55 ± 0.034	60.30 ± 0.65
Water extract	141.20 ± 0.75	0.57 ± 0.023	412.60 ± 0.47
Ascorbic acid	4.15 ± 0.24	19.28 ± 0.045	54.8 ± 0.76

**Table 2:** Antiplasmodial activity and toxicity of *S. palustris* (Burm.f.) Bedd. extracts.

Sample	Antiplasmodial (IC_50_; *μ*g/mL)	Toxicity (LC_50_; *μ*g/mL)
*n*-Hexane extract	>100	>1000
Dichloromethane extract	21.68 ± 0.56	>1000
Ethyl acetate extract	11.06 ± 0.45	>1000
Water extract	62.19 ± 0.34	>1000
Artemisinin (control)	0.0054 ± 0.0013	—

**Table 3:** Antibacterial of *S. palustris* (Burm.f.) Bedd. extracts at a concentration of 500 *μ*g/disc for Gram-negative and Gram-positive bacteria.

Microorganisms	*Inhibition zone (cm)*				
	*n*-Hexane	Dichloromethane	Ethyl acetate	Water extract	Chloramphenicol
*Bacillus subtilis*	0.00 ± 0^e^	**1.30** **±** **0.03**^**b**^	1.11 ± 0.01^c^	0.73 ± 0.02^d^	3.18 ± 0.02^a^
*Bacillus cereus*	0.00 ± 0^d^	0.85 ± 0.02^bc^	**0.90** **±** **0.01**^**b**^	0.73 ± 0.02^c^	2.57 ± 0.04^a^
*Streptococcus aureus*	0.75 ± 0.02^d^	**0.93** **±** **0.03**^**b**^	0.85 ± 0.01^c^	0.73 ± 0.02^d^	2.91 ± 0.03^a^
*Vibrio parahaemolyticus*	0.00 ± ^d^	0.86 ± ^c^	**0.93** **±**^**b**^	0.82 ±^c^	2.15 ±^a^
*Listeria monocytogenes*	0.00 ± 0^c^	0.00 ± 0^c^	**0.84** ± 0.03^**b**^	0.00 ± 0^c^	2.51 ± 0.05^a^
*Salmonella typhi*	0.00 ± 0^c^	0.82 ± 0.02^b^	**0.83** ± 0.02^**b**^	0.77 ± 0.01^b^	2.67 ± 0.03^a^
*Escherichia coli*	0.00 ± 0^b^	0.00 ± 0^b^	0.00 ± 0^b^	0.00 ± 0^b^	2.95 ± 0.01^a^

^a–e^means in the same row with different lowercase letters differed significantly (*p* < 0.05). **Bold:** the highest inhibition on each indicator microorganism.

## Data Availability

The datasets used and/or analyzed during this study are included in the article. Further clarification can be obtained from the corresponding author.
